# Psychometric properties and norm scores of the sleep self report in Dutch children

**DOI:** 10.1186/s12955-018-1073-x

**Published:** 2019-01-16

**Authors:** L. M. H. Steur, M. A. Grootenhuis, C. B. Terwee, S. Pillen, N. G. J. Wolters, G. J. L. Kaspers, R. R. L. van Litsenburg

**Affiliations:** 1 Department of Pediatric Oncology, Amsterdam UMC, Emma Children’s Hospital, Vrije Universiteit, PO BOX 7057, 1007 MB Amsterdam, The Netherlands; 2 Princess Máxima Center for Pediatric Oncology, Heidelberglaan 25, 3584 CS Utrecht, The Netherlands; 3 Department of Epidemiology and Biostatistics and the Amsterdam Public Health Research Institute, Amsterdam UMC, Vrije Universiteit, PO BOX 7057, 1007 MB Amsterdam, The Netherlands; 4 Center for Sleep Medicine, Kempenhaeghe, PO BOX 61, 5590 AB Heeze, The Netherlands; 5 Department of Pediatrics, Center for Sleep Medicine ZGT, PO BOX 546, 7550 AM Hengelo, The Netherlands

**Keywords:** Sleep self-report, Psychometric properties, Norm scores, Sleep questionnaire, Child

## Abstract

**Background:**

Psychometrically robust questionnaires to assess self-reported sleep problems in children are important since sleep problems can have a major impact on child development. The Sleep Self Report (SSR) is a 26-item self-report tool measuring different sleep domains in children aged 7–12 years. This study aims to evaluate the psychometric properties of the SSR and to provide Dutch norm scores.

**Methods:**

Children aged 7–12 years from the general population were recruited through a professional market research agency. In this population, structural validity was assessed with confirmatory and exploratory factor analyses, internal consistency was assessed with the Cronbach’s alpha coefficient and norm scores were provided. Additionally, children attending outpatient sleep clinics (clinical population) were invited to participate. SSR scores of the general population and the clinical population were compared to establish discriminative validity.

**Results:**

In total, 619 children (mean age: 9.94 ± 1.72 years) from the general population and 34 children (mean age: 9.21 ± 1.63 years) from sleep clinics participated. The 1-factor structure of the SSR was not confirmed with factor analysis. Exploratory analyses did also not yield an appropriate multidimensional structure. Internal consistency of the total score was adequate (Cronbach’s alpha: 0.76). The total score distinguished the clinical population from the general population (39.07 ± 5.31 versus 31.61 ± 5.31; *P* < 0.01).

**Conclusions:**

An appropriate structure of the SSR was not found with factor analyses in this Dutch population. The adequate internal consistency indicates that the total score can be interpreted as a measure of overall sleep problems. The SSR also shows good discriminative validity. We recommend the total score to assess overall sleep problems and item scores to evaluate specific sleep issues and to follow up children’s sleep longitudinally, as opposite changes in different item scores may not reflect in the total score. Further research on the development of multidimensional psychometrically sound pediatric sleep self-reports is of major importance.

## Background

Sleep disturbances are often reported during childhood. There is increasing evidence that sleep disturbances can negatively influence physical and psychological development in children. Sleep problems correlate, among others, with behavioral problems, poorer school performances, anxiety symptoms and depressive symptoms. [1–7] Furthermore, impaired sleep influences eating habits and may subsequently play a role in childhood overweight and obesity. [8]

Considering the importance of sleep in childhood development, it is important that sleep disturbances can be detected appropriately. In order to recognize sleep disturbances in children, valid and reliable screening tools are essential. Nowadays a variety of measures is available to measure sleep habits and sleep disturbances in children. Besides objective measures, like polysomnography and actigraphy, subjective (parent-)proxy questionnaires are often used. Previous studies reported that (parent-)proxy questionnaires do not always correlate with child self-reports. [3, 9, 10] For example, sleep onset delay and night awakenings may be assessed more appropriately by child self-reports and objective sleep measures since parents may be unaware of problems within these sleep domains, especially in older children. Therefore, when evaluating sleep in children it seems reasonable that besides objective sleep measurements and (parent-)proxy reports, child self-reports should also be taken into account.

The Sleep Self Report (SSR) is a child self-report questionnaire that was developed and validated in the United States of America for children aged 7–12 years. [9] It is a 26-item, one week retrospective survey which was designed to assess different sleep constructs, like “bedtime resistance” and “daytime sleepiness”. The structure of the SSR was developed based on the Children’s Sleep Habits Questionnaire (CSHQ), a (parent-)proxy survey that was developed for children 4–10 years. The CSHQ has already been validated in Dutch children and Dutch norms are available. [9, 11, 12]

Factor analysis of the SSR in the original American population yielded 23-items for a total SSR score, reflecting the constructs sleep duration, bedtime resistance, night awakenings, and daytime sleepiness. Internal consistency was considered good in this population with a Cronbach’s alpha of 0.88. [9] However, results of the factor analysis with fit statistics were not presented. Furthermore, other psychometric qualities such as discriminative validity, to reflect the differentiating ability of the questionnaire, were not determined in this population. [9] Aside from the original population, the psychometric properties of the SSR have only been evaluated in a Spanish and German study. [13, 14] In the Spanish sample of school-aged children, a 4-factor structure based on 16 items was extracted measuring the constructs sleep quality, sleep anxiety, bedtime refusal and sleep routines. [13] Internal consistency was good for the 16 item total score and adequate for three out of the four extracted subscales. [13] Based on these results the authors recommended the use of the SSR by clinicians and researchers for the assessment of sleep in Spanish school-aged children. [13] In the German study, the internal consistency of the total score was good with a Cronbach’s alpha of 0.71 and re-test reliability was moderate (*r* = 0.51). [14] In addition, they reported the questionnaire was well able to differentiate between a community sample and a clinical sample. [14].

However, other thorough psychometric evaluations of the SSR have not previously been performed. Furthermore, according to the report by Gozal and Spruyt in 2011, multidimensional sleep self-report questionnaires are scarce and none of the available self-report questionnaires fulfilled all the eleven steps of tool development. [15] Therefore, this study aims to investigate the psychometric properties (structural validity, internal consistency, and discriminative validity) of the SSR in children aged 7–12 years. Dutch norm scores were collected as representative Dutch norm scores are not available.

## Methods

### Participants and procedures

Data collection in the general population was conducted as part of a large Dutch study aiming at establishing norm scores for a set of questionnaires. In January 2016, children aged 7–12 years and their parents who were fluent in Dutch were invited by e-mail to participate in the study. Participants were blinded to the constructs being measured by the questionnaires to minimize the risk for selection bias. The online data collection was conducted in cooperation with the Taylor Nelson Sofres Netherlands Institute for Public Opinion (TNS NIPO), a Dutch market research agency. The TNS NIPO provides access to respondents in the TNS NIPObase. The TNS NIPObase is a database with a panel of 55.000 households who have indicated to be willing to participate in TNS NIPO research on a regular basis. The TNS NIPO uses the software program ‘DIANA’ for sampling and weighing procedures [16]. The sample was stratified based on Dutch population figures regarding key demographics (age, sex, marital status and educational level). A stratified random sampling technique was used to minimize sample variance and to increase precision. Based on an expected response rate of around 60 %, a stratified sample of 1016 children aged 7–12 years and their parents was drawn from the panel in order to obtain 100 respondents per year of age. A sample size of 100 participants per calendar year was aimed to be able to perform factor analyses. In addition, with 100 participants per calendar year we were able to present norm scores for each age group.

Respondents received a minimal financial compensation based on the length of the questionnaires. The entire general population was recruited in order to represent the general Dutch population. The subgroup of children without a chronic illness and without sleep medication use represented healthy children that are not at increased risk of sleep difficulties.

Additionally, to provide a clinical population, children referred to an outpatient sleep clinic (of a general hospital) with any kind of sleep problem were included between November 2015 and June 2017. Children without mental impairments who were able to complete the questionnaire themselves were invited to participate during the first visit at the clinic. Questionnaires were completed on paper in the clinical population. A sample size between *n* = 23 and *n* = 86 was aimed based on data of a previous Dutch norm study including a small sample of children who completed the SSR. [3] The mean total SSR score and standard deviation (SD) in this population were 37.8 and 13.4 respectively. A difference of 0.5–1 SD between the general and clinical population was considered relevant. To demonstrate a difference of 0.5 SD or 1 SD a sample size of n = 86 or n = 23, respectively, was needed.

In the general population informed consent was obtained by the TNS NIPO. In the clinical population parents and children that were 12 years of age provided written informed consent. The study was approved by the Institutional Review Board of the VU University medical center Amsterdam and the Academic Medical Center Amsterdam.

### Measures

The SSR is a one-week retrospective self-report sleep survey, which was developed for children aged 7–12 years. It assesses different sleep domains, like bedtime resistance, daytime sleepiness and sleep duration. [9] The SSR consists of 26-items and allows for a total score based on 23-items in the original population of American children. Children are asked to indicate the frequency of occurrence of these 23-items in the most recent, typical week. A three point scale is used, consisting of: usually (5–7 times a week), sometimes (2–4 times a week) or rarely (0–1 times a week). Answers are converted to a score ranging from 1 to 3. Some of the items are reversely scored. These items were converted so that a higher score indicates more sleep problems for all items. The three remaining questions are not represented in the total score but provide additional information with regards to bedtime routines. The self-report survey is developed based on the structure and items of the CSHQ, a multidimensional (parent)proxy survey assessing sleep in children aged 4–10 years. [9] Thirteen SSR items directly correlate with CSHQ items. The Dutch translated version of the SSR was used in this study. [3]

In the general cohort, children were required to complete each question online before they were able to continue to the next question, therefore no items were missing. In the clinical population missing items were imputed, by item means of the clinical population, if less than half of the items on a scale were missing.

General information about the child’s gender, child’s age and parents’ educational level was provided by TNS NIPO or the parents in clinical population. The family’s socio-economic status (SES) was classified based on the highest completed parental education, stratified according to the classification of “Volksgezondheidenzorg.info”. [17] This organization is part of the Dutch National Institute for Health and Environment and publishes scientifically substantiated information about public health in the Netherlands based on information of Statistics Netherlands. [18] According to this classification SES was divided in 3 levels: low, intermediate and high. In addition, parents in the general and clinical population provided information on whether or not their child had any chronic mental and/or physical disorders and/or used sleep medication.

### Statistical analysis

The key demographic variables (child age, child sex and SES) of responders and non-responders within the general population were compared to assess whether a representative sample was recruited. Furthermore, demographic variables of the responders of the general population and the clinical population were compared. Independent samples T-tests were used for comparison of continuous variables between two groups. Chi-square tests or Fishers Exact tests were used for categorical variables.

Structural validity was assessed with factor analyses. Confirmatory Factor Analysis (CFA) was performed in the entire general cohort to determine whether the original one-factor structure could be replicated. The CFA was then repeated in two different age-groups: 7–9 years olds and 10–12 year olds. As children completed the questionnaires at home, without the assistance of a research team member, we hypothesized that the level of cognitive development in the younger children might impede their understanding of the questions. This might have influenced the factor structure of the questionnaire in younger children. Furthermore, sleep behaviors are age dependent and age differences in factor structures have previously been described in literature. [11, 12, 19]

The clinical 3-factor structure, as determined by the domains assessed (bedtime resistance, sleep behavior (sleep duration and night awakenings), and daytime sleepiness), was also evaluated using CFA in the entire general cohort. This was not performed in the separate age groups because our hypothesis was not confirmed with the CFA of the single factor structure in the separate age groups. The method of weighted least squares with mean and variance adjustment was used for CFA. The Comparative Fit Index (CFI), Tucker-Lewis Index (TLI) and the Root Mean Square Error of Approximation (RMSEA) were used as measures of model fit. A CFI and TLI > 0.95 and a RMSEA of < 0.06 were considered adequate. [20] Because of a poor to moderate fit, an Exploratory Factor Analysis (EFA) was performed with Promax rotation to investigate whether another factor structure would fit in this population.

Internal consistency was assessed using the Cronbach’s alpha coefficient. A Cronbach’s alpha coefficient of 0.70–0.90 is generally considered as appropriate depending on the number of items and the sample size. [21, 22]

The SSR scores of the clinical population and the general population were compared to assess discriminative validity. Additionally, within the general population the SSR scores of children with and without a chronic disorder and children with and without use of sleep medication were compared, as children with a chronic disorder (such as, asthma, epilepsy, attention deficit hyperactivity disorder (ADHD), autism) were expected to be more at risk for the development of sleep problems. [10, 23–26] Depending on the distribution of data, Independent samples T-tests or Mann-Whitney U tests were used for comparison of SSR scores between two groups.

Norm scores (item and total scores) were presented for the entire general cohort, as a representation of the Dutch population. In addition, norm scores were presented for the subgroup of children without a chronic disorder and without sleep medication use separately, in order to represent norm scores for healthy children that are not at increased risk of sleep difficulties. [26] Furthermore, SSR scores were presented for age, sex and SES subgroups. Independent samples T-tests were used for comparison of SSR scores between two subgroups. ANOVA was used for multi-group comparison.

Statistical analyses were performed using IBM Statistics SPSS version 22. Mplus version 7 was used for factor analyses. A two-sided *p*-value of < 0.05 was considered statistically significant.

## Results

### Demographics

Sociodemographic information and SSR scores were available for 619 children in the general population (response rate 61%). Of these children 476 did not have a chronic physical and/or mental disorder neither used sleep medication, representing healthy children without increased risk for sleep difficulties. In the clinical population, 34 children completed the questionnaires. The response rate for the clinical cohort could not be calculated as the number of distributed questionnaires was not registered. Table 1 presents sociodemographic information of the general population and the clinical population. In the general population responders were not significantly different from non-responders with regards to age, sex and SES (Table 1). Sociodemographic information of the non-responders of the clinical population was not available.

**Table 1:** Demographic variables of responders and non-responders of the general population and responders of the clinical population

	General population			Clinical population	
	Responders (*n* = 619) Mean (SD)/N (%)	Non-responders (*n* = 397) Mean (SD)/N (%)	*p*-value^a^	Responders (*N* = 34) Mean (SD)/N (%)	*p*-value^b^
*Child age in years*	9.9 (1.7)	10.1 (1.7)	0.16	9.2 (1.6)	0.02
*Female sex*	285 (46.0)	182 (45.8)	0.95	14 (41.2)	0.58
*Socio-economic status*			0.54		0.14
- Low	83 (13.4)	46 (11.6)		4 (11.8)	
- Middle	293 (47.3)	184 (46.3)		11 (32.4)	
- High	240 (38.8)	166 (41.8)		19 (55.9)	
- Missing	3 (0.5)	1 (0.3)		-	
*Chronic physical and/or mental disorder*	138 (22.3)	N/A	N/A	22 (64.7)^c^	< 0.01
*Use of sleep medication*	11 (1.8)	N/A	N/A	12 (35.3)	< 0.01

*SD* Standard deviation, *N/A* Not available

^a^*p*-value for difference between responders and non-responders

^b^*p*-value for difference between responders in the norm population and the clinical population

^c^percentage based on number of complete cases for that item

The sex distribution in the general cohort (46% female) was comparable to Dutch population numbers (49% female) in the same age group. [27] Less than 15% of the families was stratified as a low SES in our general cohort while in the Dutch population 30% is stratified as low SES according to Statistics Netherlands. [28] In the general cohort of this study 47% of the families were classified as intermediate SES and 39% as high SES, compared to 39 and 30% in the Dutch population respectively.

The prevalence of children that were reported to have any chronic physical and/or mental disorder was somewhat higher compared to the prevalence in the Dutch population in children aged 4–12 years (22.3% versus 14%). [29] In addition, in a previous Dutch study based on general practitioner contacts and referrals, a prevalence of 12.6% was found in children 0–14 years of age. [30] However, in previous Dutch norm studies, in the same age group, comparable numbers (20.2–26%) were reported by parents. [31, 32] The prevalence of sleep medication use in the general cohort (1.8%) was comparable to the prevalence of sleep medication and tranquilizers use in the Dutch population in children aged 0–12 years (1.0–5.9%). [33]

Children in the clinical population were significantly younger compared to the responders of the general population. However, the mean age difference was less than a year. As expected, children in the clinical population were significantly more often indicated as having a chronic physical and/or mental disorder and using sleep medication compared to the responders of the general population. Furthermore, families in the clinical population were more often classified as high SES (56%) compared to families in the general population (39%), although the difference was not statistically significant.

### Factor analyses

CFA of the original single factor structure did not fit well, with moderate to poor fit statistics. CFI was 0.79, TLI 0.77 and RMSEA 0.07. Five items (“go to bed at the same time every night”, “fall asleep in the same bed every night”, “fall asleep in parents’, brothers’ or sisters’ bed”, “brings a special thing to bed (doll, blanket etc)”, “thinks he/she sleeps too much”) were subsequently deleted because of low factor loadings. However, fit statistics did not improve significantly. CFI was 0.83, TLI was 0.81 and RMSEA 0.08. In the separate age groups the original single factor structure with 23 items also did not fit well, with moderate to poor fit statistics in both groups (7–9 years olds: CFI 0.87, TLI 0.85, RMSEA 0.07; 10–12 year olds: CFI was 0.82, TLI 0.80, RMSEA 0.08).

The 3-factor structure was also not appropriate in the entire general cohort, even after deleting the 3 items with the lowest factor loadings (“go to bed at the same time every night”, “brings a special thing to bed (doll, blanket etc)”, “thinks he/she sleeps sleep too much”). CFI was 0.81, TLI was 0.79 and RMSEA was 0.06.

EFA was subsequently performed in the entire general cohort, with a fixed number of factors ranging from 2 to 5 factors. However, EFA did not yield a more appropriate factor structure.

### Internal consistency

Internal consistency of the total score based on 23 items was considered appropriate with a Cronbach’s alpha of 0.76.

### Discriminative validity

Significantly more sleep problems were found in the clinical population (total score on 23 items: mean 39.07; SD 5.31) compared to the general population (mean 31.61; SD 5.31; *p* < 0.01). Within the general population a statistically significant higher SSR score was found in children with a chronic disorder and children that used sleep medication compared to children without a chronic disorder and without sleep medication use (Table 2).

**Table 2:** Total Sleep Self Report scores of the general population (*n* = 619)

	Total SSR score Mean (SD)/Median [IQR]	*p*-value
*Child age*		0.84
- 7 years (*n* = 104)	31.13 (4.94)	
- 8 years (*n* = 104)	32.05 (5.46)	
- 9 years (*n* = 104)	31.89 (5.92)	
- 10 years (*n* = 105)	31.66 (5.39)	
- 11 years (*n* = 103)	31.37 (5.43)	
- 12 years (*n* = 99)	31.58 (4.66)	
*Child sex*		0.64
- Male	31.52 (5.26)	
- Female	31.72 (5.37)	
*Socio-economic status*		0.39
- Low	31.16 (4.66)	
- Intermediate	31.47 (5.46)	
- High	31.96 (5.37)	
*Chronic disorder*		< 0.01
- Yes	32.67 (5.66)	
- No	31.31 (5.17)	
*Sleep medication*		< 0.01
- Yes	38.0 [31.0;42.0]	
- No	31.0 [28.0;35.0]	

*SSR* Sleep Self Report, *SD* Standard deviation, *IQR* Interquartile range

### Norm scores

The SSR total score did not differ significantly between age, sex and SES subgroups (Table 2). Table 3 presents norm scores of the total general population as well as for the subpopulation of children without a chronic physical or mental disorder and/or sleep medication use.

**Table 3:** Item and total Sleep Self Report scores of the general population

	Total general population (*n* = 619) Mean (SD)/n(%)	General population without children with a chronic disorder and/or sleep medication use (*n* = 476) Mean (SD)/n(%)
*Who in your family sets the rules about when you go to bed?*		
- (One of the) parents	606 (97.9)	464 (97.5)
- The child	6 (1.0)	6 (1.3)
- Together	3 (0.5)	3 (0.6)
- Other	4 (0.6)	3 (0.6)
*The child self-reports trouble sleeping*	129 (20.8)	79 (16.6)
*The child likes to go the bed*	464 (75.0)	364 (76.5)
**23 items for the total score**		
Do you go to bed at the same time every night on school nights?*	1.24 (0.47)	1.26 (0.49)
Do you fall asleep in the same bed every night?*	1.10 (0.31)	1.10 (0.31)
Do you fall asleep alone?*	1.10 (0.38)	1.09 (0.34)
*Do you fall asleep in parents’, brothers’ or sisters’ bed?*	1.15 (0.47)	1.15 (0.46)
Do you fall asleep within 20 min?*	1.64 (0.77)	1.57 (0.75)
*Do you fights with your parents about going to bed?*	1.31 (0.55)	1.29 (0.54)
*Is it hard for you to go to bed?*	1.55 (0.68)	1.51 (0.64)
Are you ready for bed at your usual bedtime?*	1.57 (0.71)	1.54 (0.71)
*Do you have a special thing (doll, blanket* etc.*) you bring to bed?*	2.37 (0.89)	2.37 (0.89)
*Are you afraid of the dark?*	1.55 (0.75)	1.52 (0.73)
*Are you afraid of sleeping alone?*	1.25 (0.56)	1.23 (0.53)
*Do you stay up late when your parents think you are asleep?*	1.43 (0.63)	1.42 (0.62)
*Do you think you sleep too little?*	1.40 (0.60)	1.38 (0.57)
*Do you think you sleep too much?*	1.13 (0.42)	1.14 (0.43)
*Do you wake up at night when your parents think you’re asleep?*	1.31 (0.52)	1.28 (0.51)
*Do you have trouble falling back to sleep if you wake up during the night?*	1.41 (0.64)	1.40 (0.63)
*Do you have nightmares?*	1.25 (0.47)	1.24 (0.45)
*Does pain wake you up at night?*	1.10 (0.31)	1.09 (0.30)
*Do you sometimes go to someone’s bed during the night?*	1.09 (0.31)	1.08 (0.29)
*Do you have trouble waking up in the morning?*	1.78 (0.75)	1.79 (0.75)
*Do you feel sleepy during the day?*	1.38 (0.57)	1.35 (0.55)
*Do you take naps during the day?*	1.02 (0.16)	1.02 (0.17)
*Do you feel rested after a night’s sleep?*	1.47 (0.68)	1.43 (0.65)
**Total score**	31.61 (5.31)	31.27 (5.12)

*SD* standard deviation, * Reversely scored items

## Discussion

It is important to have psychometrically robust questionnaires available to assess self-reported sleep problems in children as sleep problems can have a major impact on child development. In this study the psychometric properties of the Dutch version of the SSR were evaluated and Dutch norm scores were provided. The data collection was conducted by the TNS NIPO, guaranteeing a robust and representative sample with an equal distribution of respondents (around 100 children) among age years. Based on the results of this study the SSR can be recommended to assess overall sleep problems in Dutch children aged 7–12 years. However, we could not apply a multidimensional structure of the questionnaire in this population.

The CFA yielded poor to moderate fit statistics indicating that the original one-factor structure was not appropriate in Dutch children aged 7–12 years. Our hypothesis that the factor structure could be age dependent, due to the development of sleep behaviors during childhood and the differences in level of cognitive development, was not confirmed in this population. The original single factor structure could not be replicated in either of the age subgroups. The original 23-item one-factor structure was established by Owens et al. in American children aged 7–12 years. [9, 34] However, the fit statistics of the factor analysis were not presented. [9] Owens et al. also reported on an 13-itemed SSR total score in American children with ADHD. [10] However, these items were selected based on their correlation with the CSHQ instead of on psychometric evaluations. [10]

As the questionnaire assesses multiple dimensions of sleep, a one-factor structure, measuring a single construct may not be the appropriate structure for this questionnaire. However, CFA of the 3-factor structure, based on the domains assessed by the questionnaire, also did not fit. Furthermore, based on the results of the EFA, with only minimal changes of the questionnaire a multiple factor structure assessing different sleep constructs could also not be applied to our sample. In contrast to our results, Orgiles et al. reported a multiple factor structure in their Spanish sample based on the EFA results. A 4-factor structure was extracted with an explained variance of 46% and a good fit with CFA. However, this 4-factor structure was based on only 16 appropriate items. [13] Furthermore, cultural differences between our population and the Spanish population may explain the contrasting results as cultural differences are known to influence sleep. [35]

Although the SSR total score may not be suited to measure a well-defined single sleep construct it can still be interpreted as a measure of overall sleep problems, based on the 23 items assessing several sleep domains, as was confirmed by the good internal consistency. Clinicians and researchers should be aware of the fact that fluctuations in the SSR total score over time could be based on changes in different sleep domains. Even so, opposite changes in different item scores may not be reflected in the total score. Furthermore, between groups, differences in the total score can be caused by variations in different sleep domains in each group. As an appropriate subscale structure did not apply to our population, item scores can be considered as an alternative for follow up over time and for comparison between groups on specific sleep issues.

The Cronbach’s alpha of the total score, based on 23 items, was above 0.70 in our population as well as in the original American population and the Spanish population. [9, 13] In the German population a Cronbach’s alpha of above 0.70 was reported based on 29 items. [14] The appropriate internal consistency in our population as well as in the American and the Spanish population support the application of the 23 items for a total score to measure overall self-reported sleep problems in children. The internal consistency of the 13 item total score, that was suggested in American population of children with ADHD and that was also used in a previous Dutch population, was not determined in these populations. [3, 10] As the 13-itemed total score was only based on the correlation with the CSHQ it was not assessed in the current study.

With respect to the discriminative validity, children referred to an outpatient sleep clinic indeed reported more sleep problems compared to children in the general population. Furthermore, within the general population more sleep problems were reported by children with a chronic disorder and children that used sleep medication. These results suggests that the SSR is able to adequately differentiate between children with and without sleep problems as it is known from literature that children with certain comorbidities are more at risk for the development of sleep problems. [10, 23–26] As was expected children referred to an outpatient sleep clinic were more likely to suffer from a chronic disorder and to use sleep medication. The presence of a comorbidity in these children may be associated with the reason for referral to a sleep clinic. [10, 25]

This study has some limitations. Firstly, the sample size of the clinical population was relatively small, however, still a statistically significant difference was found between the clinical and general population.

Secondly, the SES classification was based on highest parental educational level. Only the educational level of the parent with the highest level of education was considered and therefore SES could have been overestimated. Furthermore, current household income was not taken into account. In the general cohort of this study families with a higher SES were overrepresented compared to the Dutch population. Furthermore, in the clinical cohort more families with a high SES were represented compared to the general cohort. As it is known from literature that educational level, often used as a proxy for SES, correlates with sleep, this might have influenced our results. [36–39] Results on the direction of the association between educational level and sleep outcomes are, however, not always consistent. A lower educational level was sometimes associated with more sleep problems such as, excessive daytime sleepiness and insomnia. [36, 37] In contrast, in the 2013 study by Gardner et al. those with the lowest educational level were reported to have fewer problems with, among others, falling asleep, sleep maintenance and daytime sleepiness compared to those with the highest educational attainment. [38] In the current study, however, the impact of the SES distribution is expected to be limited as no differences were found in the SSR total score between SES subgroups within our general study population.

Thirdly, the prevalence of children that were reported to have any chronic physical and/or mental disorder was somewhat higher in our general cohort compared to the prevalence in the Dutch population. [29, 30] The difference in age range between the populations may account for a part of the difference in prevalence. Furthermore, in our population the prevalence of a chronic illness was parent reported, which might have overestimate the prevalence of a chronic illness. As was confirmed by the similar prevalence’s found in other parent reported studies. [31, 32]

Fourthly, children in the clinical population were somewhat younger compared to the norm population. However, as the age difference between groups was only minimal the clinical relevance of this difference will probably be negligible. Furthermore, as age differences in SSR total score within the norm population were not found it seems unlikely that this have impacted our results.

Finally, the questionnaires were administrated differently in the general population and the clinical population. The data collection was conducted online in the general population whereas paper-and-pencil questionnaires were completed in the clinical population. This might have influenced the difference in total score between the general population and the clinical population. However, in a previous meta-analysis electronic measures were reported to be equivalent to paper-and-pencil measures. [40] Furthermore, the good discriminative validity was confirmed between subpopulations within the general population.

The results of this study are generalizable to the Dutch population. In previous Dutch and Portuguese norm studies differences in norm scores between countries were found. [3, 41] Although there are similarities in child sleep across countries, child sleep behaviors can be influenced by cultural habits. Therefore, we recommend to use cultural specific norm scores.

Although a variety of sleep measurements for children are available nowadays, thoroughly investigated multidimensional self-reports are scarce. Spruyt and Gozal reported in a review that questionnaire development should include eleven essential steps, from a research question (step 1), to the generation of items (step 4), the validation (step 9) and the standardization and collection of norm scores (step 11). [42] In 2011, they summarized for which of the available sleep questionnaires, both proxy- and self-reports, the 11 steps of validation and standardization were conducted [15]. None of the self-report questionnaires fulfilled all the 11 steps in tool development. Two questionnaires fulfilled 7 (Insomnia Scale) and 8 (Pediatric Daytime Sleepiness Scale) of the steps. Both questionnaires only concerned a single construct (daytime sleepiness and insomnia) and norm scores were not available for both questionnaires (step 11). Unfortunately, the SSR was not mentioned separately. Only the CSHQ, fulfilling 5 of the 11 steps, was reported. They recommended the development of universally adopted and validated self-report tools for future research. [15]

## Conclusion

In conclusion, the SSR can be used to assess overall sleep problems. Researchers and clinicians are recommended to use item scores for the assessment of specific sleep issues, and to follow childrens’ sleep longitudinally as balanced changes in item scores may not reflect in the total score. Moreover, the questionnaire offers the opportunity to distinguish between children with and without sleep problems. As thoroughly assessed multidimensional sleep self-report questionnaires are still lacking it is of major importance that research to improve and develop self-report sleep tools in children will continue in the future.

## Abbreviations

ADHD: Attention Deficit Hyperactivity Disorder
CFA: Confirmatory Factor Analysis
CFI: Comparative Fit Index
CSHQ: Children’s Sleep Habits Questionnaire
EFA: Exploratory Factor Analysis
RMSEA: Root Mean Square Error of Approximation
SD: Standard deviation
SES: Socio-economic status
SSR: Sleep Self Report
TLI: Tucker-Lewis Index
TNS-NIPO: Taylor Nelson Sofres Netherlands Institute for Public Opinion
